# Human *Mycobacterium bovis* Infection and Bovine Tuberculosis Outbreak, Michigan, 1994–2007

**DOI:** 10.3201/eid1404.070408

**Published:** 2008-04

**Authors:** Melinda J. Wilkins, Joshua Meyerson, Paul C. Bartlett, Susan L. Spieldenner, Dale E. Berry, Laura B. Mosher, John B. Kaneene, Barbara Robinson-Dunn, Mary Grace Stobierski, Matthew L. Boulton

**Affiliations:** * Michigan Department of Community Health, Lansing, Michigan, USA; † Health Department of Northwest Michigan, Charlevoix, Michigan, USA; ‡ Michigan State University, East Lansing, Michigan, USA; § Beaumont Hospital, Royal Oak, Michigan, USA; ¶ University of Michigan, Ann Arbor, Michigan, USA

**Keywords:** Mycobacterium bovis, bovine tuberculosis, deer, cattle, humans, dispatch

## Abstract

*Mycobacterium bovis* is endemic in Michigan’s white-tailed deer and has been circulating since 1994. The strain circulating in deer has remained genotypically consistent and was recently detected in 2 humans. We summarize the investigation of these cases and confirm that recreational exposure to deer is a risk for infection in humans.

Historically, *Mycobacterium bovis* infection in humans was associated with consumption of unpasteurized milk and dairy products (*1*,*2*) and this is still the most important route of exposure in developing countries. US populations are exposed to unpasteurized dairy products imported from countries where *M. bovis* is prevalent (*3*,*4*). *M. bovis* infection in humans is of concern to health officials in Michigan because of to its endemicity in the state’s wild white-tailed deer population and its discovery in several cattle herds. *M. bovis* in deer represents possible occupational and recreational routes of exposure to humans, especially for hunters, trappers, taxidermists, venison processors, and venison consumers (*5*).

Although *M. bovis* is a zoonotic agent, surveillance indicates no increase in its incidence in Michigan residents since an outbreak began in 1994. Since 1995, the incidence rate of *M. bovis* infection in Michigan residents has remained very low, with ≈1 new case per year for a total of 13. No genetic or epidemiologic link to the deer/cattle outbreak strain has been identified among 11 of these human *M. bovis* cases, based on restriction fragment length polymorphism analysis, spoligotyping, or mycobacterial interspersed repeat units (MIRU) typing (M. Wilkins, unpub. data, Michigan Department of Community Health, March 2007). Table 1 shows the spoligotyping and MIRU typing results from 9 available human specimens that were unrelated to the deer/cattle outbreak strain. All genotyping of isolates mentioned in this report was performed at the Michigan Department of Community Health, Bureau of Laboratories, Lansing, MI, USA, by using currently recommended guidelines (Centers for Disease Control and Prevention) (*8–**10*). The remaining 2 human cases of *M. bovis* occurred in US-born, Michigan residents; the cases had epidemiologic and molecular links to the genotypically consistent deer/cattle outbreak strain circulating in Michigan.

**Table 1:** Nonepidemiologically linked human *Mycobacterium bovis* spoligotyping and MIRU typing results for 9 available human specimens, Michigan*

Country of birth	Site	Year collected	International spoligotype†	MIRU type	Spoligotypes
USA	Cervical LN	1997	SB1210	Not available	▫▪▫▪▫▫▪▪▫▫▫▫▪▪▪▫▪▪▪▪▪▪▪▪▪▪▪▪▪▪▪▪▪▪▪▪▪▪▫▫▫▫▫
USA	Spine	1997	SB0131	Not available	▪▪▫▪▫▪▪▪▫▪▫▪▪▪▪▫▪▪▪▪▪▪▪▪▪▪▪▪▪▪▪▪▪▪▪▪▪▪▫▫▫▫▫
Mexico	Abd abscess	1998	SB0140	232224152322	▪▪▫▪▪▫▪▫▫▫▫▫▪▪▪▫▪▪▪▪▪▪▪▪▪▪▪▪▪▪▪▪▪▪▪▪▪▪▫▫▫▫▫
Mexico	Sputum	1998	SB1210	Not available	▫▪▫▪▫▫▪▪▫▫▫▫▪▪▪▫▪▪▪▪▪▪▪▪▪▪▪▪▪▪▪▪▪▪▪▪▪▪▫▫▫▫▫
Bosnia	Cervical LN	2000	SB1215	232314353312	▪▪▫▪▪▪▪▫▫▫▫▫▪▫▪▫▪▪▪▪▪▪▪▪▪▪▪▪▪▪▪▪▪▪▪▪▪▪▫▫▫▫▫
Mexico	Sputum	2003	SB0121	232314223322	▪▪▫▪▪▪▪▪▫▪▪▪▪▪▪▫▪▪▪▪▫▪▪▪▪▪▪▪▪▪▪▪▪▪▪▪▪▪▫▫▫▫▫
USA	Cervical LN	2003	SB0121	232324252322	▪▪▫▪▪▪▪▪▫▪▪▪▪▪▪▫▪▪▪▪▫▪▪▪▪▪▪▪▪▪▪▪▪▪▪▪▪▪▫▫▫▫▫
Mexico	Cervical LN	2005	SB1216	232124233222	▫▫▫▪▪▪▪▪▫▪▫▪▪▪▪▫▪▪▪▪▪▪▪▪▪▪▪▫▫▫▪▪▪▪▪▪▪▪▫▫▫▫▫
Mexico	Cervical LN	2007	SB0140	231224243322	▪▪▫▪▪▫▪▫▫▫▫▫▪▪▪▫▪▪▪▪▪▪▪▪▪▪▪▪▪▪▪▪▪▪▪▪▪▪▫▫▫▫▫

*MIRU, mycobacterial interspersed repeat units (*6*); LN, lymph node; Abd, abdominal. †International Spoligotype Database, available from www.mbovis.org (*7*).

## The Cases

### Patient 1, 2002

In January 2002, a 74-year-old man sought medical care, reporting malaise, anorexia, and fever. Past medical history included ischemic bowel disease, vascular disease, partial gastrectomy for peptic ulcers, and left upper lobectomy for squamous cell carcinoma (December 1999). On February 1, he was hospitalized with persistent fever and nonproductive cough; results of a chest radiograph were consistent with necrotizing pneumonia. A tuberculosis (TB) skin test (TST) result was negative, and a sputum smear was negative for acid-fast baccilli (AFB). After 5 days, the patient had not improved clinically; chest radiograph showed increasing infiltrate on the left side. Diagnostic bronchoscopy was performed, which yielded an AFB-positive smear. The condition of the patient deteriorated clinically over the next 10 days; he died on day 16 of his hospitalization.

Laboratory confirmation for TB, speciation, and antimicrobial drug susceptibility testing were pending at the time of his death. Genotyping analysis showed that the *M. bovis* isolated from this patient matched the circulating deer/cattle strain (Table 2).

**Table 2:** Epidemiologically linked *Mycobacterium bovis* spoligotyping and MIRU typing results, Michigan*

Species	Year collected	International spoligotype†	MIRU type	Spoligotypes
Deer 1	1997	SB0145	Not available	▪▪▫▪▫▫▫▫▫▫▫▫▫▫▪▫▪▪▪▪▪▪▪▪▪▪▪▪▪▪▪▪▪▪▪▪▪▪▫▫▫▫▫
Bovine 1	1998	SB0145	Not available	▪▪▫▪▫▫▫▫▫▫▫▫▫▫▪▫▪▪▪▪▪▪▪▪▪▪▪▪▪▪▪▪▪▪▪▪▪▪▫▫▫▫▫
Bovine 2	1999	SB0145	232224253322	▪▪▫▪▫▫▫▫▫▫▫▫▫▫▪▫▪▪▪▪▪▪▪▪▪▪▪▪▪▪▪▪▪▪▪▪▪▪▫▫▫▫▫
Bovine 3	1999	SB0145	232224253322	▪▪▫▪▫▫▫▫▫▫▫▫▫▫▪▫▪▪▪▪▪▪▪▪▪▪▪▪▪▪▪▪▪▪▪▪▪▪▫▫▫▫▫
Bovine 4	2002	SB0145	232224253322	▪▪▫▪▫▫▫▫▫▫▫▫▫▫▪▫▪▪▪▪▪▪▪▪▪▪▪▪▪▪▪▪▪▪▪▪▪▪▫▫▫▫▫
Human patient 1	2002	SB0145	232224253322	▪▪▫▪▫▫▫▫▫▫▫▫▫▫▪▫▪▪▪▪▪▪▪▪▪▪▪▪▪▪▪▪▪▪▪▪▪▪▫▫▫▫▫
Deer 2	2003	SB0145	232224253322	▪▪▫▪▫▫▫▫▫▫▫▫▫▫▪▫▪▪▪▪▪▪▪▪▪▪▪▪▪▪▪▪▪▪▪▪▪▪▫▫▫▫▫
Human patient 2	2004	SB0145	232224253322	▪▪▫▪▫▫▫▫▫▫▫▫▫▫▪▫▪▪▪▪▪▪▪▪▪▪▪▪▪▪▪▪▪▪▪▪▪▪▫▫▫▫▫
Deer 3‡	2004	SB0145	232224253322	▪▪▫▪▫▫▫▫▫▫▫▫▫▫▪▫▪▪▪▪▪▪▪▪▪▪▪▪▪▪▪▪▪▪▪▪▪▪▫▫▫▫▫

*MIRU, mycobacterial interspersed repeat units (*6*). †International Spoligotype Database, available from www.mbovis.org (*7*). ‡Deer shot by patient 2.

In his youth, patient 1 lived on a farm geographically distant from the current bovine TB–endemic area. His first wife had a reported diagnosis of TB after their divorce >40 years before, and his second wife reports he drank unpasteurized milk as a youth. He moved to the edge of Deer Management Unit (DMU) 452 in 1994, which is the focal area for the bovine TB outbreak in deer. There, he ran a business with a buck pole where hunters displayed killed deer. Additional potential exposures included hunting white-tailed deer and consuming venison (>10 years before his death), handling a deer carcass from the DMU 452 vicinity in 2000, and recreational feeding of deer.

This patient was in poor health at the time of death, having acute and chronic illness. His poor health would have rendered him more susceptible to infection with *M. bovis* and would have made the progression from latent infection to clinical disease more likely. The pathology results from his lung resection in December 1999 provided no evidence of TB; therefore, infection was likely acquired subsequently. The genotyping results from patient 1 matched those of the circulating deer/cattle strain, which suggested exposure to infected cattle or deer. The lack of recent exposure to cattle suggests that deer are the more likely source of infection.

### Patient 2, 2004

Patient 2 was a 29-year-old, previously healthy man. On October 1, 2004, he shot a white-tailed deer just outside DMU 452. While field dressing the animal, he punctured his left index finger with a hunting knife. Approximately 18 days after the injury, his finger became inflamed and painful, so he sought medical treatment. Based on his history of exposure to a deer with lesions, a TST was administered; the result was negative. After ≈10 days of antimicrobial drug therapy, the wound had not improved. He was hospitalized, and the infected finger was incised and drained. An orthopedic specialist diagnosed infectious tenosynovitis of the flexor tendon of the left index finger. The initial slide preparation was negative for AFB. A wound culture was sent to Michigan Department of Community Health, Bureau of Laboratories.

The patient was discharged and then readmitted to the hospital 12 days later with subcutaneous infection at the puncture site, which was again incised and drained. A slide made of growth from the broth culture medium was positive for AFB. Genetic probe results confirmed *M. tuberculosis* complex. By December 7, 2004, the culture was reported as resistant to pyrazinamide, suggesting *M. bovis,* which was later confirmed on the basis of susceptibility to thiopene-2-carboxylic acid hydrazide and biochemical testing for pyrazinamidase. The result of a second skin test, 14 weeks postexposure, was positive (6-mm induration). He continued to receive antimicrobial drug therapy for 9 months without further complications

As an experienced hunter, patient 2 recognized the tan nodules in the deer’s chest cavity as *M. bovis* and promptly buried the carcass. In December, he led Michigan Department of Natural Resources staff back to the carcass, which was retrieved; the chest cavity was filled with lesions (Figure). Although the carcass was buried for >9 weeks, chest cavity samples were submitted for culture. After numerous attempts with alternative decontamination techniques, a viable culture was obtained. Genotyping results of the carcass isolate were identical to that recovered from patient 2 and the circulating deer/cattle strain (Table 2). The investigation of the infection in patient 2 provided strong evidence of transmission of *M. bovis* infection from deer to human through percutaneous injection with a contaminated hunting knife. The patient’s history of hunting exposure was essential to diagnosis and treatment of this rare form of TB.

**Figure:**
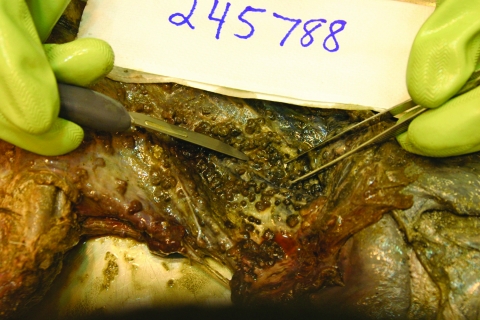
Photo of the chest cavity of a deer shot by patient 2; the deer was retrieved after being buried for 9 weeks. The photo shows the classical nodular lesions of *Mycobacterium bovis* infection. Photo: J.S. Fierke, D.J. O’Brien, S.M. Schmitt, Wildlife Disease Laboratory, Michigan Department of Natural Resources.

## Conclusions

Although epidemiologic evidence presented for patient 1 is not irrefutable, we conclude that both cases are part of a cluster that is epidemiologically and genotypically confirmed (*11*). The initial TST result was negative in both of these cases, likely due to cutaneous anergy (patient 1) and administration too soon after exposure (patient 2). Initial negative skin test results made diagnosis problematic for healthcare providers.

The confirmation of a hunter’s acquiring cutaneous *M. bovis* from an infected deer supports the need for public health precautions. First, hunters should wear heavy latex or rubber gloves while field dressing deer. Second, hunter education was important in the second case because the hunter recognized the deer as infected and specifically mentioned his exposure each time he sought medical treatment. Third, efforts to raise the index of suspicion of the medical community regarding cutaneous and other occupational or recreational exposures to TB continues to be important, so that appropriate diagnoses can be made. Finally, in both cases, the initially negative TST result complicated the diagnostic efforts. It is an ongoing challenge to ensure that providers appropriately apply and interpret the TST.
